# Clinical Significance of Plasma D-Dimer in COVID-19 Mortality

**DOI:** 10.3389/fmed.2021.638097

**Published:** 2021-05-25

**Authors:** Yayun Li, Yuhao Deng, Lin Ye, Huiyan Sun, Songtao Du, Huining Huang, Furong Zeng, Xiang Chen, Guangtong Deng

**Affiliations:** ^1^Hunan Key Laboratory of Skin Cancer and Psoriasis, Department of Dermatology, Hunan Engineering Research Center of Skin Health and Disease, Xiangya Hospital, Central South University, Changsha, China; ^2^National Clinical Research Center for Geriatric Disorders, Xiangya Hospital, Central South University, Changsha, China; ^3^Shanghai Ninth People's Hospital, Shanghai Jiao Tong University School of Medicine, Shanghai, China; ^4^Department of Colorectal Surgical Oncology, Harbin Medical University Cancer Hospital, Harbin, China; ^5^Department of Oncology, Xiangya Hospital, Central South University, Changsha, China

**Keywords:** COVID-19, SARS-CoV-2, D-dimer, independent, cutoff, meta-analysis

## Abstract

It is not clear whether D-dimer can be an independent predictor of coronavirus disease 2019 (COVID-19) mortality, and the cut-off of D-dimer for clinical use remains to be determined. Therefore, a comprehensive analysis is still necessary to illuminate the clinical significance of plasma D-dimer in COVID-19 mortality. We searched PubMed, Embase, Cochrane Library, and Scopus databases until November 2020. STATA software was used for all the statistical analyses. The identifier of systematic review registration was PROSPERO CRD42020220927. A total of 66 studies involving 40,614 COVID-19 patients were included in our meta-analysis. Pooled data showed that patients in high D-dimer group had poor prognosis than those in low D-dimer group [OR = 4.52, 95% CI = (3.61, 5.67), *P* < 0.001; HR = 2.81, 95% CI = (1.85, 4.27), *P* < 0.001]. Sensitivity analysis, pooled data based on different effect models and the Duval and Tweedie trim-and-fill method did not change the conclusions. Subgroup analyses stratified by different countries, cutoffs, sample size, study design, and analysis of OR/HR still keep consistent conclusions. D-dimer was identified as an independent predictor for COVID-19 mortality. A series of values including 0.5 μg/ml, 1 μg/ml, and 2 μg/ml could be determined as cutoff of D-dimer for clinic use. Measurement and monitoring of D-dimer might assist clinicians to take immediate medical actions and predict the prognosis of COVID-19.

## Introduction

The outbreak and spread of Coronavirus Disease 2019 (COVID-19), caused by severe acute respiratory syndrome coronavirus 2 (SARS-CoV-2), had caused a pandemic around the world (1). Though most of patients had mild symptoms, a small minority of cases suffered from acute respiratory distress syndrome (ARDS) and even death (2). As of November 28, 2020, about 60 million cases have been reported by world health organization (WHO) and included around 1.5 million deaths globally (1). Worse still, the numbers of death are persistently increasing especially in the United States, the epicenter of COVID-19 (3). Therefore, identification of the independent predictors for COVID-19 mortality is still urgent and necessary to reduce the poor outcomes.

D-dimer, a fibrinogen degradation product, consists of two covalently bound fibrin D domains, which reflect the high coagulation and enhancement of secondary fibrinolytic activity *in vivo* (4, 5). Previous studies demonstrated that D-dimer was associated with the severity of COVID-19 (6–8). Hyperinflammation and hypoxia-induced injury caused by SARS-CoV-2 infection could cause the dysfunction of endothelial cells and stimulate thrombosis and elevation of D-dimer (9). Elevated D-dimer could cause the formation of pulmonary microthrombus, deep venous thrombosis, and disseminated intravascular coagulopathy, which were associated with the poor prognosis (10–12). Nowadays, increasing studies showed that D-dimer could be used as a predictor for COVID-19 mortality (9, 13). Moreover, numerous review and meta-analyses highlighted the prognostic value of D-dimer in COVID-19 mortality (14–16). However, one of the drawbacks of these analyses was that more attention was paid to D-dimer levels between survivors and non-survivors (17, 18). Actually, the abnormal elevation of D-dimer was more valuable to reflect hemodynamic changes in clinic. In addition, these meta-analyses were based primarily on the studies using univariate analysis, and it was not clear whether D-dimers play an independent role in predicting COVID-19 mortality on admission (14, 19). Another challenge is that the cutoff of D-dimer for clinical use remains to be determined (8). From the above, a comprehensive analysis of all the published studies is still necessary to illuminate the clinical significance of plasma D-dimer in COVID-19 mortality.

To our knowledge, this is the largest meta-analysis about the association between D-dimer with COVID-19 mortality. Our study will determine its cutoff and highlight the independent prognostic value of D-dimer in COVID-19 mortality to assist clinicians to take immediate medical actions and evaluate the prognosis of COVID-19.

## Materials and Methods

### Search Strategy

Our meta-analysis was performed according to the Preferred Reporting Items for Systematic Reviews and Meta-Analyses (PRISMA) statement. The following databases were searched: PubMed, Embase, Cochrane Library, and Scopus databases, from their inception to November 2020. No language restrictions were applied. The search terms were as follows: (“Coronavirus disease 2019” OR “Coronavirus 2019” OR “COVID-19” OR “COVID19” OR “Severe acute respiratory syndrome coronavirus 2” OR “SARS-CoV-2” OR “nCoV-2019” OR “2019-nCoV” OR “Novel coronavirus”) AND (“Mortality” OR “Death” OR “Dead” OR “Fatality” OR “Non-survival” OR “Non-survivors” OR “Non-survivor” OR “Prognosis” OR “Deceased”) AND (“D-dimer” OR “Laboratory”). Three of the authors (GD, FZ, and YL) independently screened initial records, titles, abstracts, and full text articles. Disagreements were resolved by discussion. In order to avoid missing relevant articles, we also manually reviewed the reference lists of selected retrieved papers as well as the major reviews and meta-analyses. The identifier of systematic review registration was PROSPERO CRD42020220927.

### Inclusion and Exclusion Criteria

Any study reporting the relationship between D-dimer and COVID-19 mortality should be included if they met the following criteria: (1) patients were diagnosed as COVID-19; (2) dichotomous D-dimer was available to evaluate the risk of COVID-19 mortality; or (3) odds ratio (OR) or hazard ratio (HR) of the D-dimer was accessible or estimated by the provided data or Kaplan-Meier curves based on the method previously described (20, 21). Exclusion criteria were as follows: (1) patients were asymptomatic carriers of SARS-CoV-2; (2) studies with smaller sample size from the same authors or institutions; and (3) patients or studies did not fulfill the inclusion criteria.

### Data Extraction and Quality Assessment

We used Endnote X9 to exclude any duplicate and irrelevant studies in our initial search. We extracted the following basic information: first authors, publication date, country of origin, study design, cases, age, sex, cutoff of D-dimer, OR, HR, and its associated 95% confidence intervals (CI). OR and HR were extracted preferentially from multivariable analysis based on lower cutoff of D-dimer. Stratified data or interquartile range such as age were converted to mean (standard deviation) based on the mathematical formulas for meta-analysis (22, 23). We used Newcastle-Ottawa Scale (NOS) for quality assessments. Two authors (GD and FZ) independently selected and evaluated the included articles. When a consensus was lacking, a third reviewer (LY) was consulted to solve the disagreements.

### Statistical Analysis

STATA (Version 12.0; STATA Corporation, College Station, TX, USA) and TSA (Copenhagen trial unit) software were used for all the statistical analyses. OR with 95% CI was calculated for binary outcomes, and HR for time-to-event outcomes (24). Random-effect and fixed-effect models were both adopted in all analyses to assess the stability of results. Additionally, sensitivity analyses were performed by omitting one study each time; meta-regression and subgroup analyses were conducted based on different countries, cutoffs, sample size, study design, and analysis of OR/HR to further evaluate the consistency of our conclusions. The funnel plot and Egger test was used to evaluate publication bias, and the Duval and Tweedie trim-and-fill method was performed to adjust for this bias (25). Trial sequential analysis was used to eliminate early false positive findings. *P* < 0.05 was considered statistically significant.

## Results

### Literature Search and Studies Characteristics

**Figure 1:**
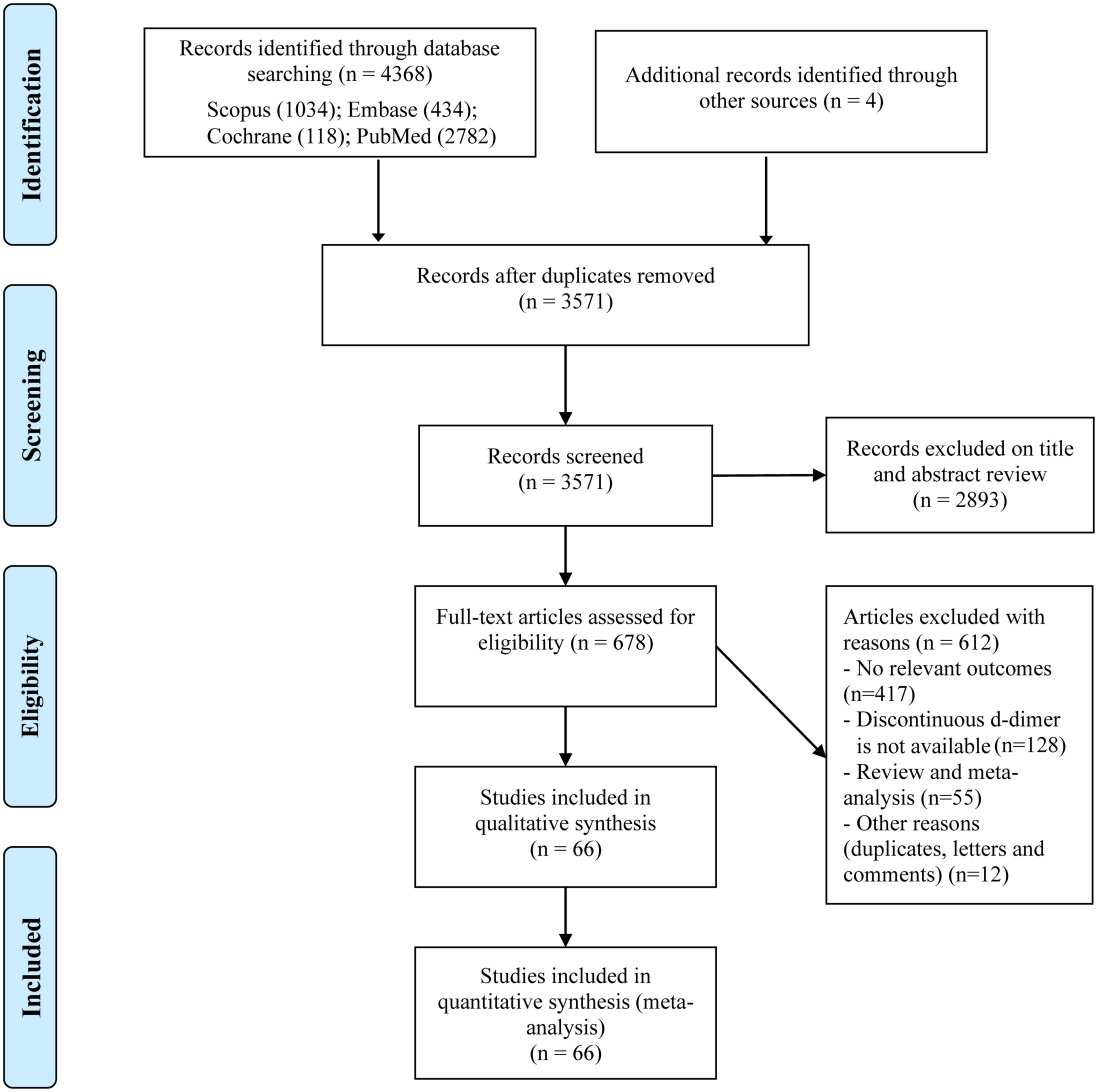
Selection flow chart of the study.

We initially identified 4,372 records through our search strategy and scanning the reference lists of related meta-analyses (Figure 1); 3,571 studies remained after excluding duplicates. Then we reviewed the titles and abstracts and obtained 678 studies for full-text scanning. We further excluded 612 studies due to studies without our concerned outcomes (*n* = 371), studies without dichotomous D-dimer (*n* = 128), review and meta-analyses (*n* = 55) and other reasons including duplicates, letters, and comments (*n* = 12). Finally, a total of 66 studies involving 40,614 COVID-19 patients were included in our meta-analysis (9, 13, 26–89).

The main characteristics of eligible studies are shown in Table 1. All these 66 studies were published in 2020 and from different countries including China, the United States, Italy, Turkey, Spain, Mexico, and Switzerland. In these studies, 65 studies were written in English, and one in Chinese, and 22 studies had sample size above 500 patients. What's more, 56 studies reported OR and 15 reported HR of D-dimer. Except one study, all studies of high quality had seven or more NOS scores, and details are shown in Supplementary Table 1.

**Table 1:** Characteristics of eligible studies.

**References**	**Country**	**Study design**	**Cases**	**Age (years)**	**Sex (male %)**	**Cutoff (μg/ml)**	**Variables**	**NOS scores**
Aloisio et al. (20)	Italy	Single-center	427	61.4 ± 17.1	293 (68.6%)	16.28	OR	7
Ayanian et al. (27)	USA	Single-center	299	–	161 (53.8%)	3	OR	7
Bahl et al. (28)	USA	Multi-center	1461	62.0 ± 17.8	770 (52.7)	0.5 1	OR	7
Barman et al. (29)	Turkey	Multi-center	607	59.5 ± 15.6	334 (55.0%)	0.5	OR^*^	8
Bazzan et al. (30)	Italy	–	88	60.7 ± 12.8	60 (68.1%)	3	OR	6
Berenguer et al. (31)	Spain	Multi-center	4035	68.6 ± 17.8	2433 (61.0%)	0.5	OR	7
Berger et al. (32)	USA	Multi-center	2377	63.3 ± 16.3	1495 (62.8%)	0.23 2	OR^*^	9
Bhargava et al. (33)	USA	Single-center	419	–	211 (50.4%)	1.5	OR	7
Cao et al. (34)	China	Single-center	102	52.6 ± 22.6	53 (52.0%)	0.5	OR	7
Chen et al. (35)	China	Multi-center	1590	48.3 ± 68.2	904 (57.3%)	0.5	OR	7
Chen et al. (36)	China	Single-center	203	55.1 ± 53	108 (53.2%)	0.5	OR	7
Chen et al. (37)	China	Single-center	274	58.5 ± 19.4	171 (62.4%)	21	OR	7
Chen et al. (38)	China	Single-center	73	65.8 ± 10.1	42 (57.5%)	2.35	OR	7
Chen et al. (39)	China	Multi-center	635	59.9 ± 14.1	318 (50.0%)	0.5	HR	7
Cheng et al. (40)	China	Single-center	305	62.5 ± 14.2	184 (60.3%)	0.845	HR	7
Chilimuri et al. (41)	USA	Single-center	375	62.3 ± 14.9	236 (62.9%)	1	OR^*^	9
Cortés-Tellés et al. (42)	Mexico	Single-center	200	53.6 ± 17.9	138 (69.0%)	0.7	OR	7
Du et al. (43)	China	Single-center	179	57.6 ± 13.7	97 (54.2%)	0.5	OR	7
Feng et al. (44)	China	Multi-center	476	52.3 ± 17.8	271 (56.9%)	1	HR^*^	8
Giacomelli et al. (45)	Italy	Single-center	233	–	72 (30.9%)	0.5 1	OR HR^*^	8
Guisado-Vasco et al. (46)	Spain	Single-center	607	69.0 ± 16.3	394 (65.0%)	2.5	OR^*^	9
Huang et al. (47)	China	Multi-center	676	54.2 ± 21.5	314 (46.4%)	0.5	OR HR^*^	8
Laguna-Goya et al. (48)	Spain	Single-center	501	52.0 ± 11.9	317 (63.3%)	1.368	OR	7
Li et al. (50)	China	Multi-center	523	53.4 ± 15.3	275 (52.6%)	1.09	HR^*^	8
Li et al. (49)	China	Single-center	2068	61.2 ± 14.1	1005 (48.6%)	0.5	OR	7
Li et al. (51)	China	Single-center	102	57.4 ± 18.8	59 (57.8%)	0.5 1	OR	7
Li et al. (52)	China	Single-center	113	67.3 ± 14.1	68(60.2%)	20	OR	7
Li et al. (53)	China	Multi-center	245	51.5 ± 20.1	118 (48.2%)	1	HR^*^	8
Li et al. (54)	China	Multi-center	132	64.3 ± 10.5	70 (53.0%)	1.5	OR^*^	8
Liao et al. (55)	China	Multi-center	380	63.3 ± 14.9	206 (54.2%)	2	OR^*^	9
Liu et al. (56)	China	Single-center	214	67.6 ± 12.7	119 (55.6%)	1	HR^*^	8
Liu et al. (57)	China	Single-center	1190	57.0 ± 14.8	635 (53.4%)	0.5 1	OR	7
Lu et al. (58)	China	Single-center	20	69.8 ± 12.0	8 (40.0%)	1	OR	7
Luo et al. (59)	China	Single-center	403	54.2 ± 21.6	193 (47.9%)	0.55 5	OR	7
Ma et al. (60)	China	Multi-center	523	43.3 ± 16.4	289 (55.3%)	0.5 1	OR	7
Manocha et al. (61)	USA	Multi-center	446	64.9 ± 15.2	291 (65.2)	6.106 6.99	OR	7
Mikami et al. (62)	USA	Multi-center	2820	65.3 ± 18.1	1611 (57.1%)	2	OR HR^*^	8
Musoke et al. (63)	USA	Single-center	355	66.2 ± 14.2	181 (51.0%)	1.5	OR^*^	9
Pan et al. (64)	China	Single-center	124	68.0 ± 10.5	85 (68.5%)	3.06	OR	7
Paranjpe et al. (65)	USA	Multi-center	1078	74.7 ± 58.7	627 (58.1%)	2	OR	7
Peng et al. (66)	China	Multi-center	49	63.0 ± 15.3	32 (65.3%)	0.5	OR	7
Petrilli et al. (67)	USA	Multi-center	2741	62.6 ± 17.1	1678 (61.2%)	2.5	HR	7
Piñana et al. (68)	Spanish	Multi-center	244	56.3 ± 64.1	132 (54.1%)	0.5	OR	7
Qin et al. (69)	China	Single-center	118	63.1 ± 15.7	49 (41.5%)	0.5 1	OR	7
Quintana-Díaz et al. (70)	Spanish	Single-center	3373	62.4 ± 23.0	1725 (48.9%)	0.5	OR^*^	8
Singh et al. (71)	USA	Single-center	276	61.6 ± 17.1	130 (47.1%)	1.18	OR	7
Song et al. (72)	China	Multi-center	248	63.4 ± 9.7	128 (51.6%)	0.5	OR	7
Tu et al. (73)	China	Single-center	174	53.0 ± 19.5	69 (39.7%)	0.5	OR	7
Volo et al. (74)	Italy	Single-center	23	64.7 ± 33.2	21 (91.3%)	4	OR	7
Wang et al. (75)	China	Single-center	548	58.7 ± 15.7	279 (50.9%)	1	OR	7
Wang et al. (76)	China	Single-center	213	60.6 ± 13.4	95 (44.6%)	0.55	OR	7
Wendel Garcia et al. (77)	Switzerland	Multi-center	639	62.3 ± 13.4	447 (75·1%)	1.5	HR^*^	8
Xia et al. (78)	China	Single-center	81	66.7 ± 11.4	54 (66.7%)	5 21	OR	7
Xie et al. (79)	China	Single-center	140	58.2 ± 15.7	72 (51.4%)	0.45	HR^*^	8
Xu et al. (80)	China	Multi-center	703	46.1 ± 15.2	382 (54.3%)	0.5	OR	7
Yang et al. (81)	China	Multi-center	203	59.9 ± 14.9	115 (56.7%)	1	OR^*^	8
Yang et al. (82)	China	Multi-center	205	63.0 ± 10.5	96 (46.8%)	0.5	OR	7
Yao et al. (83)	China	Single-center	108	48.8 ± 15.8	43 (39.8%)	1	OR	7
Yao et al. (84)	China	Single-center	248	63.0 ± 13.4	135 (54.4%)	2	OR^*^	8
Yu et al. (85)	China	Single-center	1464	61.9 ± 14.8	736 (50.3%)	0.5	OR^*^	9
Zhang et al. (86)	China	Multi-center	289	55.6 ± 49.2	154 (53.3%)	0.5	OR	7
Zhang et al. (9)	China	Single-center	343	59.5 ± 15.6	169 (49.3%)	0.5 2	OR HR	7
Zhang et al. (87)	China	Multi-center	828	60.6 ± 13.4	447 (53.99%)	1	HR	7
Zhou et al. (13)	China	Multi-center	191	56.4 ± 15.7	119 (62.3%)	0.5 1	OR^*^	8
Zhou et al. (88)	China	Single-center	67	70.6 ± 6.9	22 (32.8%)	median high	OR^*^	9
Zhou et al. (89)	China	Single-center	220	58.4 ± 16.4	104 (47.3%)	0.43 1	OR	7

*OR, odds ratio; HR, hazard ratio;*

* *Variables are calculated by multivariable analysis*.

### Association of D-Dimer and COVID-19 Mortality

**Figure 2:**
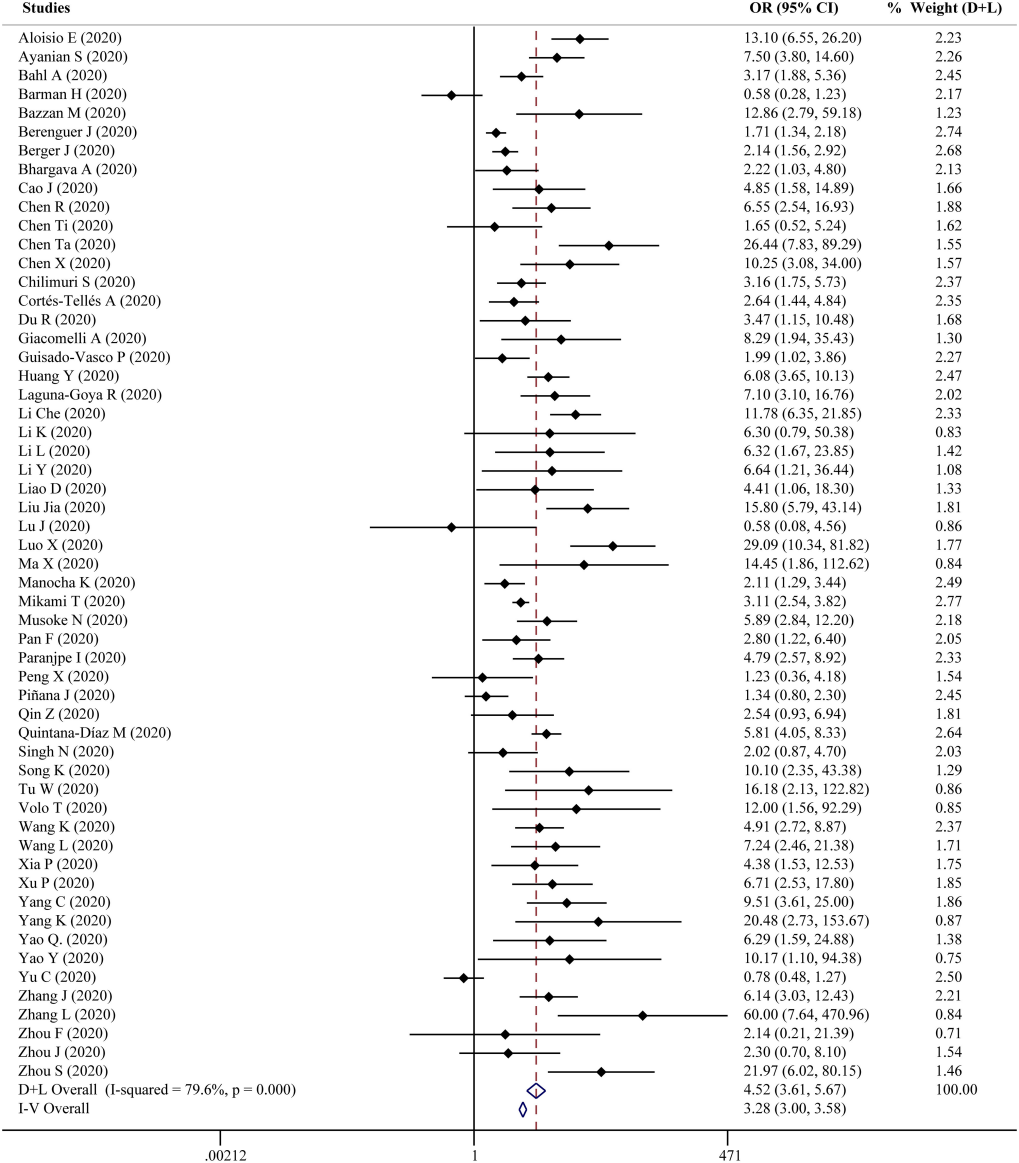
Forest plot to assess odds ratio (OR) of COVID-19 mortality for D-dimer.

**Figure 3:**
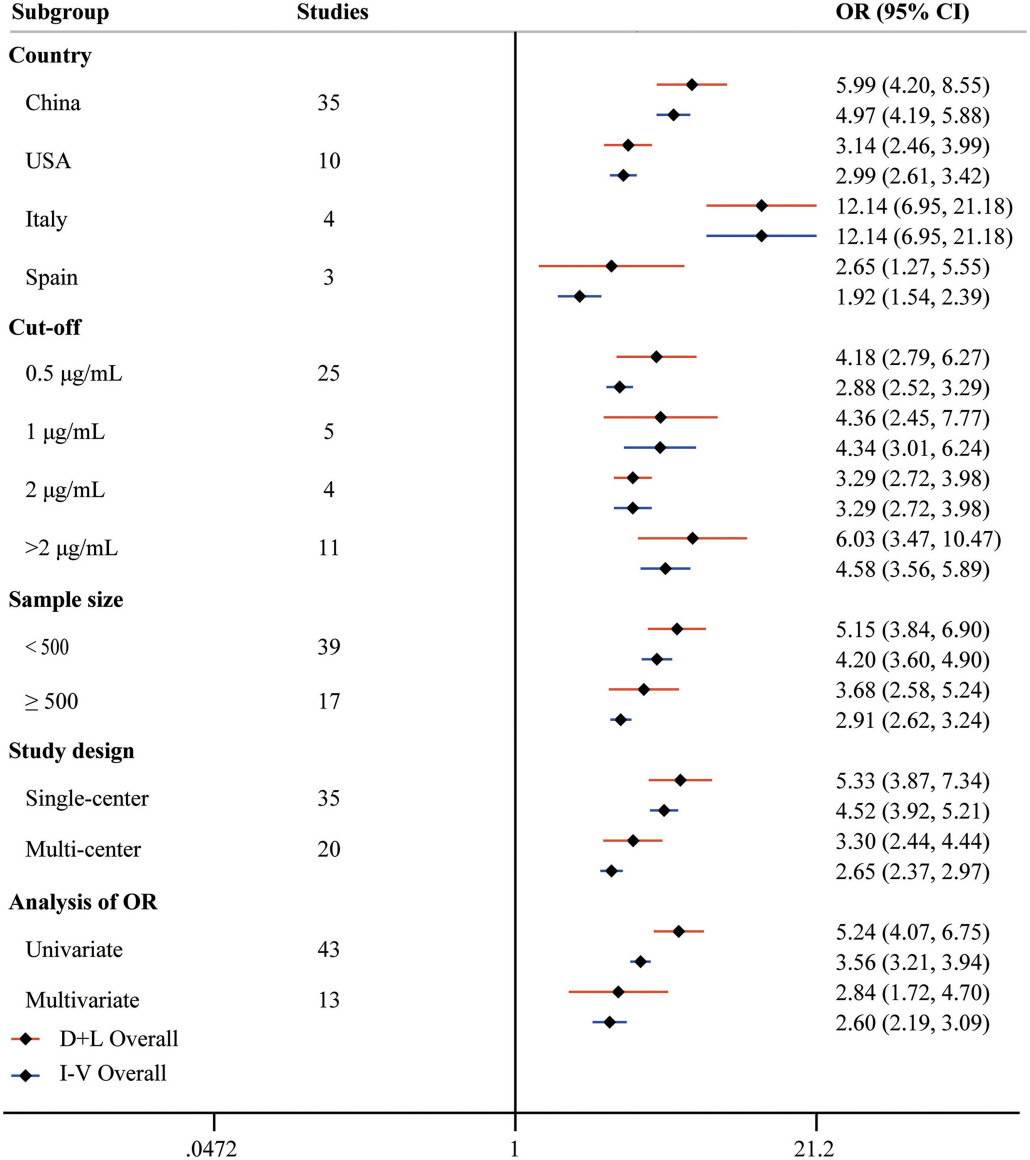
Forest plot to assess OR of COVID-19 mortality for D-dimer stratified by different countries, cutoffs, sample size, study design, and analysis of OR.

Fifty-six studies reported the proportion of non-survivors between high and low D-dimer groups. With heterogeneity (*I*^2^ = 79.6%, *P* < 0.001), the random-effect model was performed and suggested that patients in the high D-dimer group had higher proportion of mortality than those in the low D-dimer group [OR = 4.52, 95% CI = (3.61, 5.67), *P* < 0.001]. The conclusion did not change when using the fixed-effect model for meta-analysis [OR = 3.28, 95% CI = (3.00, 3.58), *P* < 0.001] (Figure 2). Sensitivity analysis did not change the conclusion (Supplementary Figure 1). The funnel plot was not in a form of symmetry, indicating the existence of potential publication bias (Supplementary Figure 2A). Then we used Egger test to detect the presence of publication bias (*P* < 0.001) (Supplementary Figure 2B). However, the conclusion did not change in fixed-effect model [OR = 2.92, 95% CI = (2.68, 3.18), *P* < 0.001] or random-effect model [OR = 3.33, 95% CI = (2.66, 4.16), *P* < 0.001] after filling 15 studies in the comparison. To examine whether the observed heterogeneity could be contributed by possible moderators, univariate meta-regression was performed and suggested that country and analysis of OR were possible significant moderators (Table 2). To further assess the stability of the conclusion, we conducted the subgroup analysis stratified by different countries, cutoffs, sample size, study design, and analysis of OR. The conclusion did not change, highlighting the independent prognostic value of D-dimer and that the cutoff of D-dimer could be determined as a series of values including 0.5 μg/ml, 1 μg/ ml, and 2 μg/ ml (Figure 3).

**Table 2:** Univariate meta regression of odds ratio (OR) of COVID-19 mortality for D-dimer.

**Variables**	**β**	**95% LCI**	**95% UCI**	***P***
**Country**				
China	0.561747	0.34901	0.904156	**0.018**
USA	1.572451	0.874163	2.828536	0.128
Italy	0.373506	0.131622	1.059905	0.064
Spain	1.743824	0.651697	4.666158	0.262
**Cut-off**				
0.5 μg/ml	1.228279	0.74308	2.030291	0.416
1 μg/ ml	1.112854	0.458536	2.700866	0.81
2 μg/ ml	1.049783	0.393859	2.798072	0.921
>2 μg/ ml	0.713175	0.381889	1.331852	0.283
Sample size	0.718948	0.430974	1.199346	0.202
**Study type**				
Single-center	0.686701	0.415319	1.135414	0.14
Multi-center	1.548838	0.937437	2.558998	0.086
Analysis of OR	0.531366	0.303191	0.931262	**0.028**

**Figure 4:**
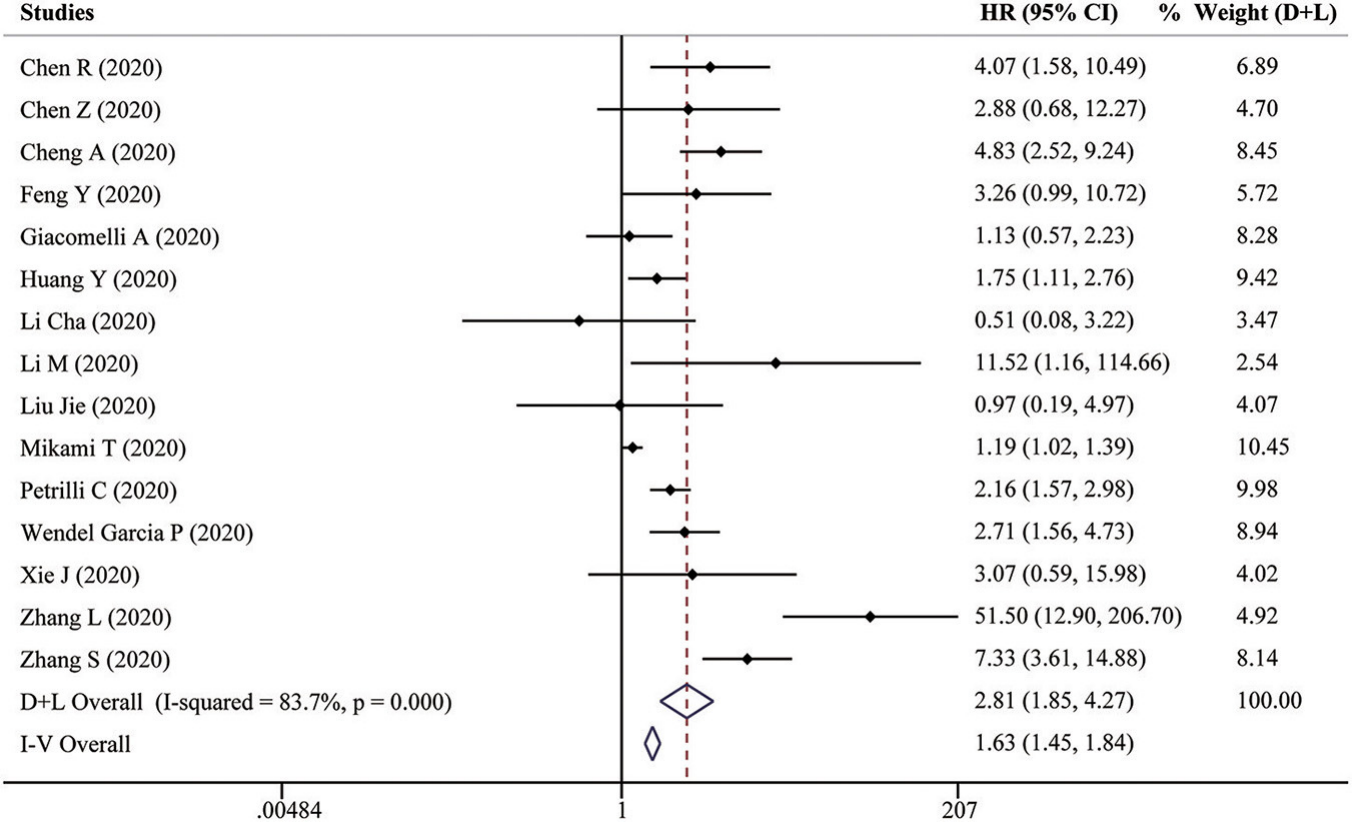
Forest plot to assess hazard ratio (HR) of COVID-19 mortality for D-dimer.

**Figure 5:**
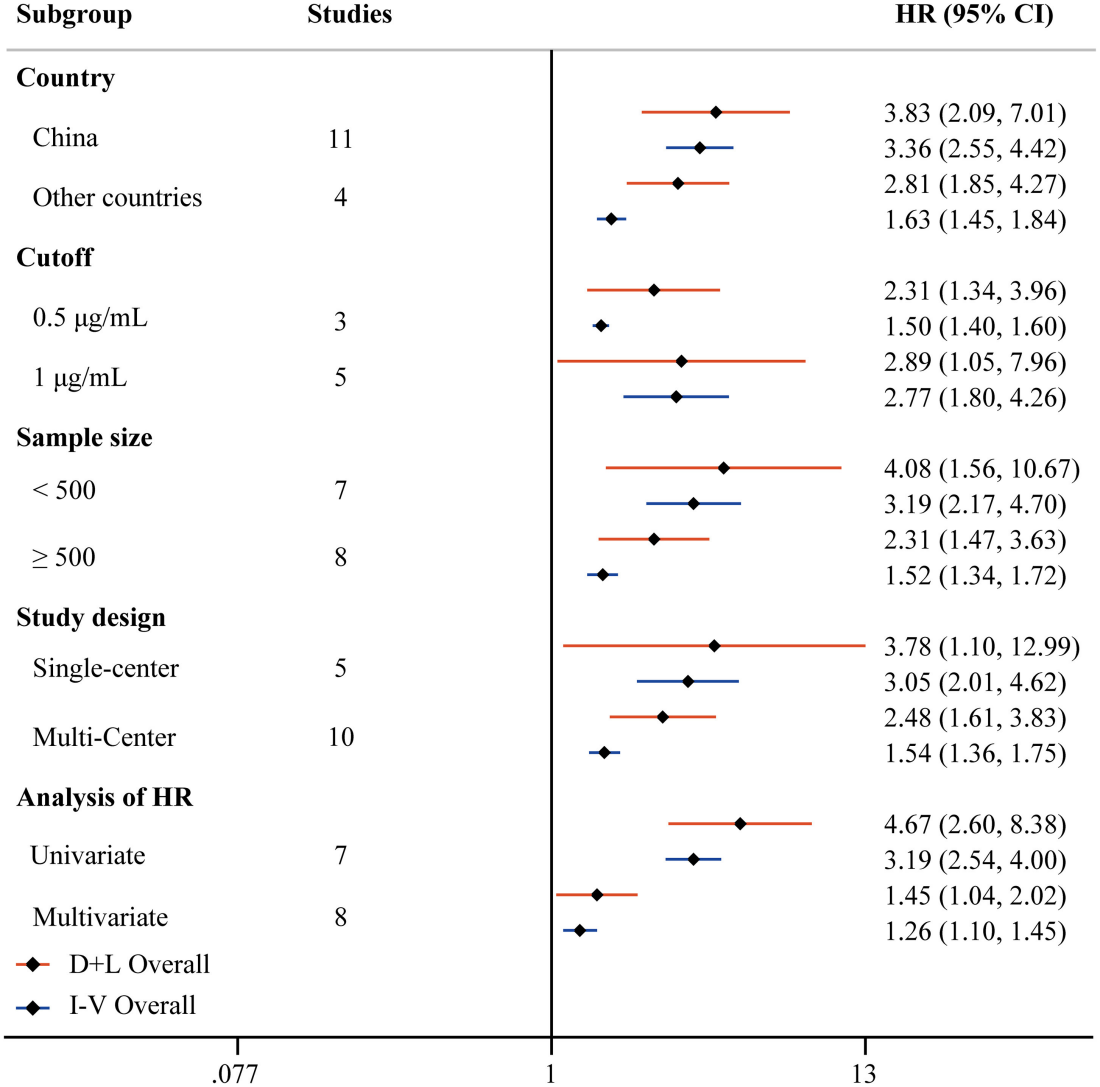
Forest plot to assess HR of COVID-19 mortality for D-dimer stratified by different countries, cutoffs, sample size, study design, and analysis of HR.

Fifteen studies reported HRs of high D-dimer vs. low D-dimer. Due to the heterogeneity among studies (*I*^2^ = 83.7%, *P* < 0.001), the random-effect model was used, and pooled data showed that patients in the high D-dimer group were significantly associated with poor overall survival [HR = 2.81, 95% CI = (1.85, 4.27), *P* < 0.001]. This result was consistent when using the fixed-effect model to analyze the pooled data [HR = 1.63, 95% CI = (1.45, 1.84), *P* < 0.001] (Figure 4). We further performed a sensitivity analysis through excluding any one specific study each time. We did not observe obvious decline of heterogeneity, and the conclusion was consistent (Supplementary Figure 3A). The funnel plot identified four studies over the pseudo 95% CI (Supplementary Figure 3B), and the Egger test detected the presence of publication bias (*P* = 0.013) (Supplementary Figure 3C). Then the Duval and Tweedie trim-and-fill method was adopted, but no studies were trimmed and filled. To explore the origin of heterogeneity, we performed the univariate meta-regression and found that analysis of HR was possible significant moderator (Table 3). Subgroup analysis based on different countries, cutoffs, sample size, study design, and analysis of HR did not change the conclusion, which means D-dimer is an independent indicator for COVID-19 mortality, and the cutoff of D-dimer (0.5μg/ ml or 1μg/ ml) could be used clinically (Figure 5).

**Table 3:** Univariate meta regression of hazard ratio (HR) of COVID-19 mortality for D-dimer.

**Variables**	**β**	**95% LCI**	**95% UCI**	***P***
Country	0.436659	0.150542	1.266569	0.117
**Cut-off**				
0.5 μg/ml	1.119004	0.263079	4.759676	0.869
1 μg/ml	1.001067	0.275978	3.631211	0.999
Sample size	0.587985	0.18532	1.865568	0.339
Study type	0.697132	0.198794	2.444712	0.545
Analysis of HR	0.351424	0.142391	0.867322	**0.027**

### Trial Sequential Analysis

**Figure 6:**
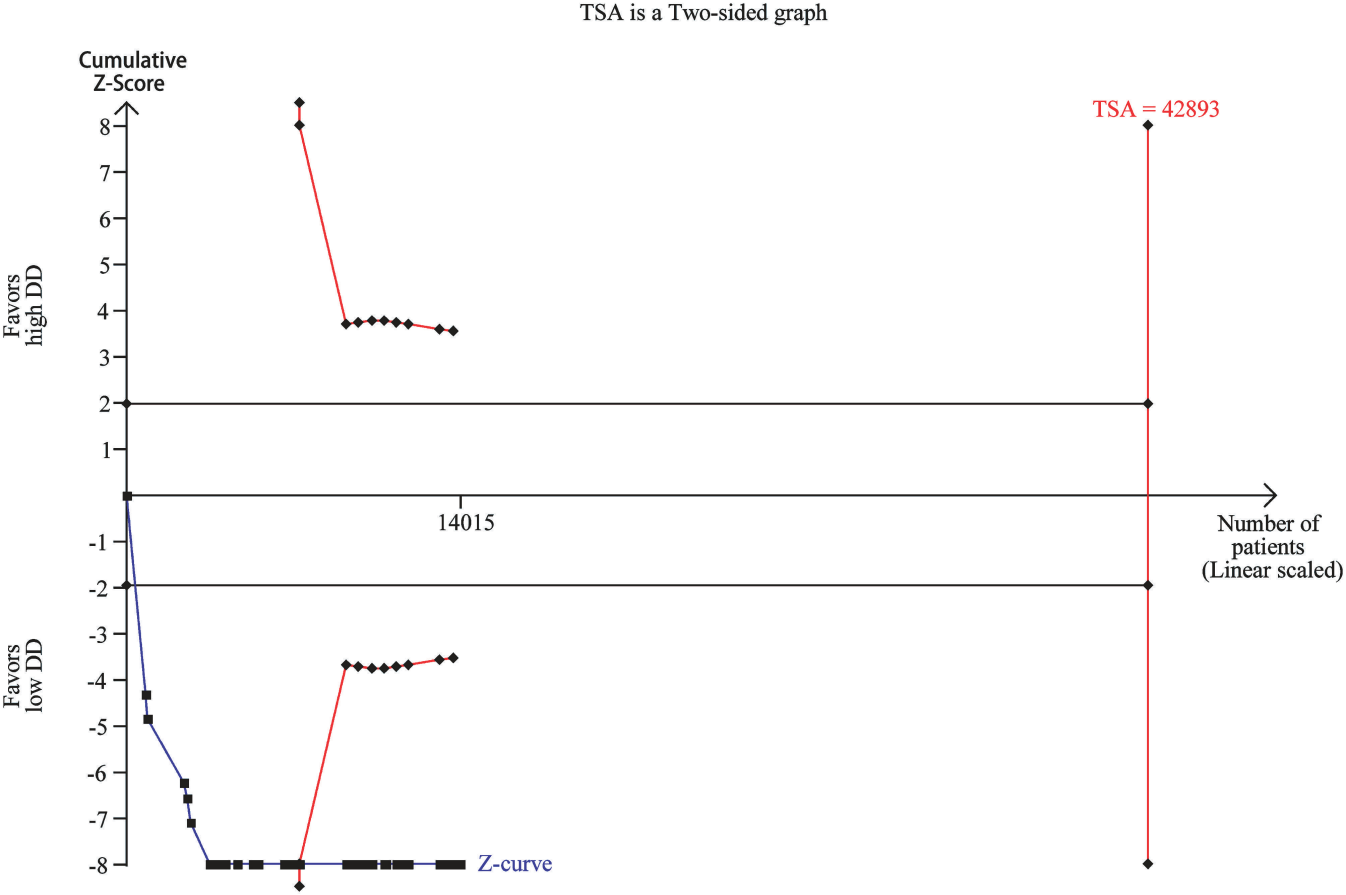
Trail sequential analysis of COVID-19 mortality between high and low D-dimer group.

Trial sequential analysis has been widely used to improve the reliability of conclusion and eliminate early false positive findings due to imprecision and repeated significance testing in meta-analyses (90). We collected the numbers of death and total numbers of patients in the high and low D-dimer group from 37 studies (Supplementary Table 2). Trial sequential analysis on data for death supported a 20% risk ratio reduction in the low D-dimer group compared with high D-dimer group. The required information size of 42,893 was calculated based on a control event proportion of 11.5% (based on data in our meta-analysis), a risk of type I error of 5%, a power of 80%, and a diversity of 87.16%. Although the actual information size did not reach the required information size, the cumulated Z-curve (blue curve) crossed the traditional boundary of 5% significance (horizontal red line) and the trial sequential monitoring boundary (red curve), implying that firm evidence was reached (Figure 6).

## Discussion

The ongoing spread of COVID-19 is posing a huge threat to global public health. Nowadays, the numbers of deaths caused by COVID-19 are still increasing while there is still no effective medication (64). Thus, it's imperative to identify the predictors for COVID-19 mortality. With regard to the role of plasma D-dimer in COVID-19 mortality, studies have reported associations that vary in strength and direction. Therefore, a comprehensive meta-analysis is necessary to illuminate the clinical significance of plasma D-dimer in COVID-19 mortality.

In this meta-analysis, a total of 66 studies involving 40,614 COVID-19 patients were enrolled. We found that patients in high D-dimer group had a poorer prognosis than those in low D-dimer group, independent of countries, cutoffs, sample size, study design, and analysis of OR/HR. Sensitivity analysis and pooled data based on different effect models were used to explore the consistency of our conclusions, and the conclusions were still consistent. Additionally, even though there exist publication bias in the combined outcomes of high D-dimer vs. low D-dimer, the conclusion still did not change after the Duval and Tweedie trim-and-fill method. Trial sequential analysis further confirmed our conclusions. Based on the above findings, we could conclude that D-dimer was an independent predictor for COVID-19 mortality. Furthermore, subgroup analyses based on cutoffs of dimer highlighted that a series of values including 0.5 μg/ ml, 1 μg/ ml, and 2 μg/ ml could be determined as the cutoff of D-dimer for clinic use.

D-dimer is one of the commonest laboratory findings for COVID-19 patients. As early as February 2019, Guan et al. reported that severe patients had a significantly higher level of D-dimer than non-severe patients through analyzing 1,099 patients with laboratory-confirmed COVID-19 from over 550 hospitals in China (91). Moreover, Zhou and his colleague conducted a retrospective study involving 191 COVID-19 patients and found that elevated D-dimer at admission was a risk factor for death of adult patients (13). However, this conclusion was not consistent in other studies. Xie et al. found that D-dimer is not a risk factor after adjustment of age and gender through analyzing 140 COVID-19 patients (79). Besides, Liu and his team even did not find the difference in the unadjusted association between D-dimer and all-cause death in COVID-19 patients (35). Therefore, our findings are necessary to solve the problem and highlight the clinical significance of plasma D-dimer in COVID-19 mortality.

The mechanism is still unknown about the association between elevated D-dimer with COVID-19 mortality. Wang et al. previously showed that the significantly increased D-dimer and corresponding hypoxemia could induce the formation of pulmonary microthrombus in the 2009 novel influenza A(H1N1) (10). A recent study conducted by Klok and his colleague demonstrated that approximately 31% COVID-19 patients in intensive care unit had the thrombotic complications (11). Moreover, D-dimer could be used to indicate deep venous thrombosis in COVID-19 patients with cardiovascular diseases (92). That means elevated level of D-dimer, the indicator of thrombotic complications, might be the cause of COVID-19 mortality. However, other studies held different opinions that COVID-19 progress is the cause of the increase of D-dimer level. One possible mechanism is that SARS-CoV-2 infections are usually accompanied by an aggressive inflammatory response and even cytokine storm. The hyperinflammation could induce the dysfunction and damage of endothelial cells, resulting in the elevation of D-dimer and excess thrombin generation (93). Additionally, organ damage and corresponding hypoxemia caused by SARS-CoV-2 infection could stimulate thrombosis through increasing blood viscosity and activating hypoxia-inducible transcription factor-dependent signaling pathways (94, 95). Recently, Turagam et al. found that mortality is mostly associated with pulseless electrical activity. Whether D-dimer-associated thrombosis could cause pulseless electrical activity and ultimately mortality needs to be clarified (96). Overall, The underlying mechanism is unsolved about the relationship between elevated D-dimer and COVID-19 mortality. Our finding highlights the association, and more studies are needed to dig out the detailed mechanism.

To our knowledge, this is the largest meta-analysis to evaluate the clinical significance of plasma D-dimer in COVID-19 mortality. However, several limitations must be acknowledged. First, noticeable heterogeneity exists in all of the analyses. Sensitivity analysis pooled the data based on different effect models, yet the heterogeneity could not be eliminated completely. Second, publication bias exists in all the comparisons, though the conclusion did not change through the Duval and Tweedie trim-and-fill method. Finally, our study could not clarify the underlying mechanism between D-dimer with COVID-19 mortality.

In conclusion, D-dimer was identified as an independent predictor for COVID-19 mortality. A series of values including 0.5 μg/ ml, 1 μg/ ml, and 2 μg/ ml could be determined as cutoff of D-dimer for clinic use. Measurement and monitoring of D-dimer might assist clinicians to take immediate medical actions and predict the prognosis of COVID-19.

## Data Availability Statement

The original contributions presented in the study are included in the article/Supplementary Material, further inquiries can be directed to the corresponding author/s.

## Author Contributions

YL and YD: data curation, formal analysis, methodology, roles/writing-original draft. LY: conceptualization, data curation, formal analysis, methodology, roles/writing-original draft. HS: formal analysis, methodology, roles/writing-revised draft. SD and HH: methodology, software, validation. FZ, GD, and XC: conceptualization, data curation, formal analysis, software, supervision, validation, visualization, roles/writing—original draft, writing—review & editing. All authors contributed to the article and approved the submitted version.

## Conflict of Interest

The authors declare that the research was conducted in the absence of any commercial or financial relationships that could be construed as a potential conflict of interest.

## Supplementary Material

Supplementary Figure 1 Sensitivity analyses of OR of COVID-19 mortality for D-dimer.

Supplementary Figure 2 Funnel plot **(A)** and Egger test **(B)** of OR of COVID-19 mortality for D-dimer.

Supplementary Figure 3 Sensitivity analyses **(A)**, funnel plot **(B)** and Egger test **(C)** of HR of COVID-19 mortality for D-dimer.

Supplementary Table 1 Methodological quality of studies included in the meta-analysis.

Supplementary Table 2 Raw data for all the analyses in the study.
