# Supplementary figures and images for: Clinical Significance of Plasma D-Dimer in COVID-19 Mortality
Source: Front Med (Lausanne). 2021 May 25;8:638097. doi: 10.3389/fmed.2021.638097 (PMC8185282; doi:10.3389/fmed.2021.638097)

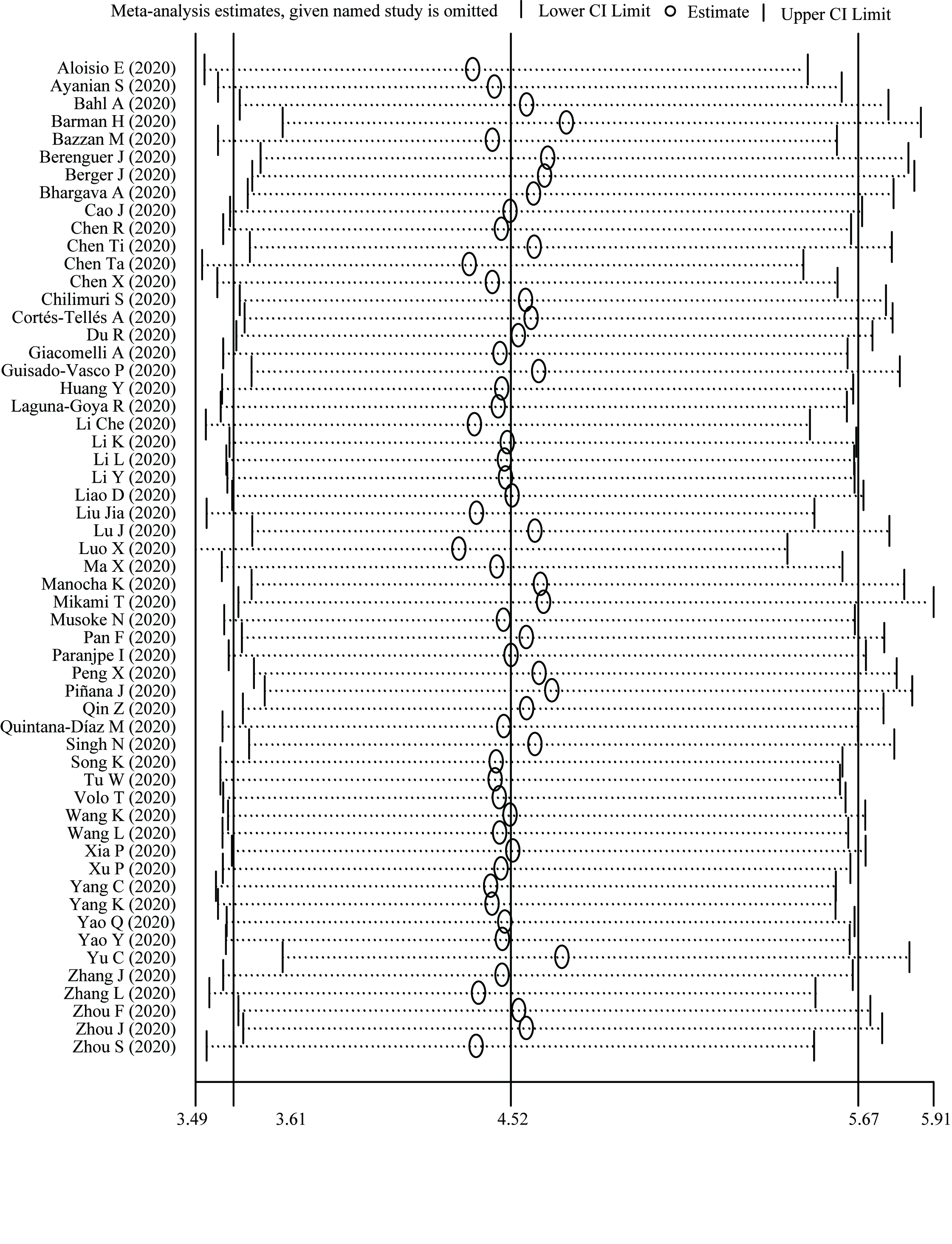

Supplement: Supplementary Figure 1 — Sensitivity analyses of OR of COVID-19 mortality for D-dimer. [file Image_1.TIF]

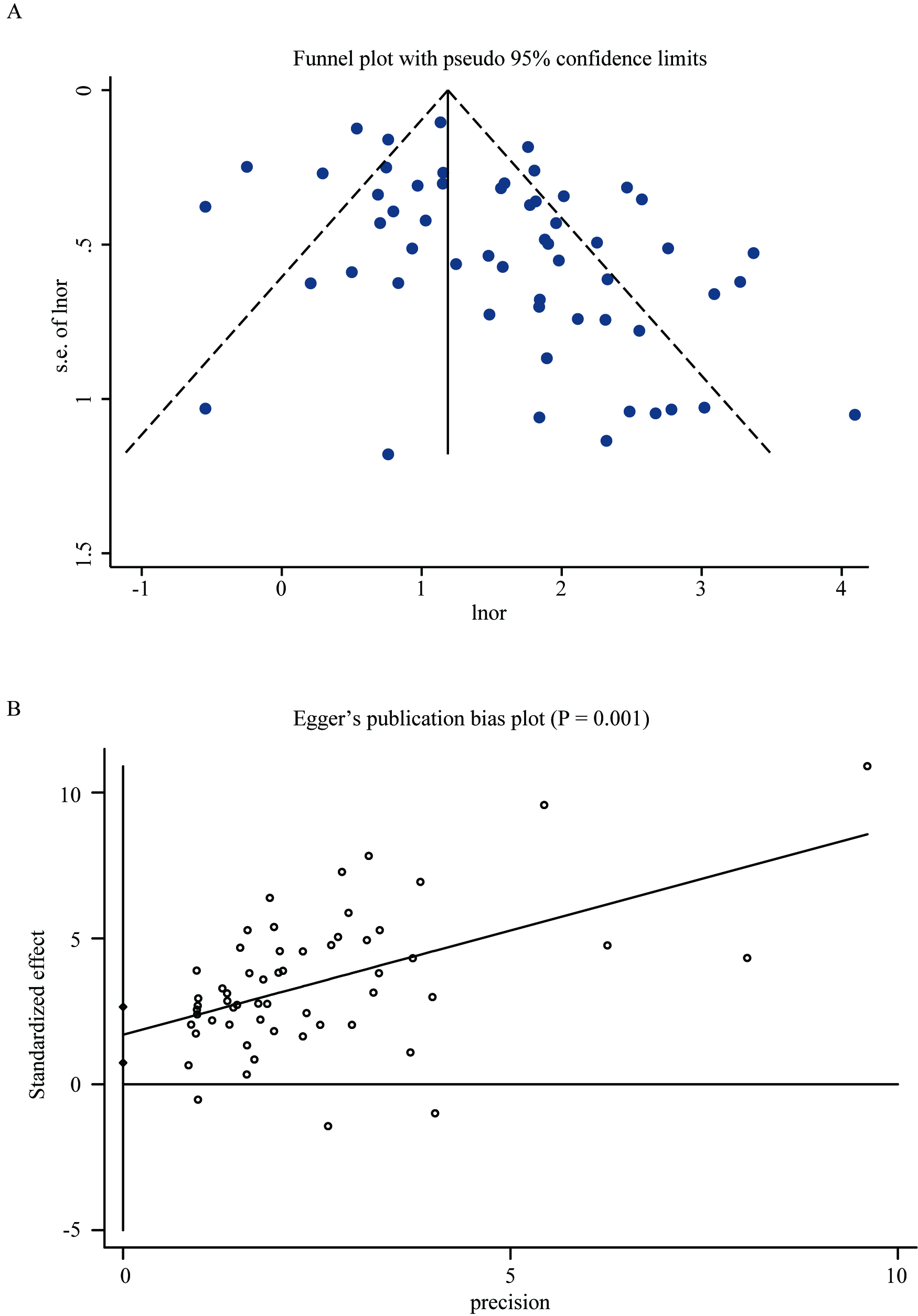

Supplement: Supplementary Figure 2 — Funnel plot (A) and Egger test (B) of OR of COVID-19 mortality for D-dimer. [file Image_2.TIF]

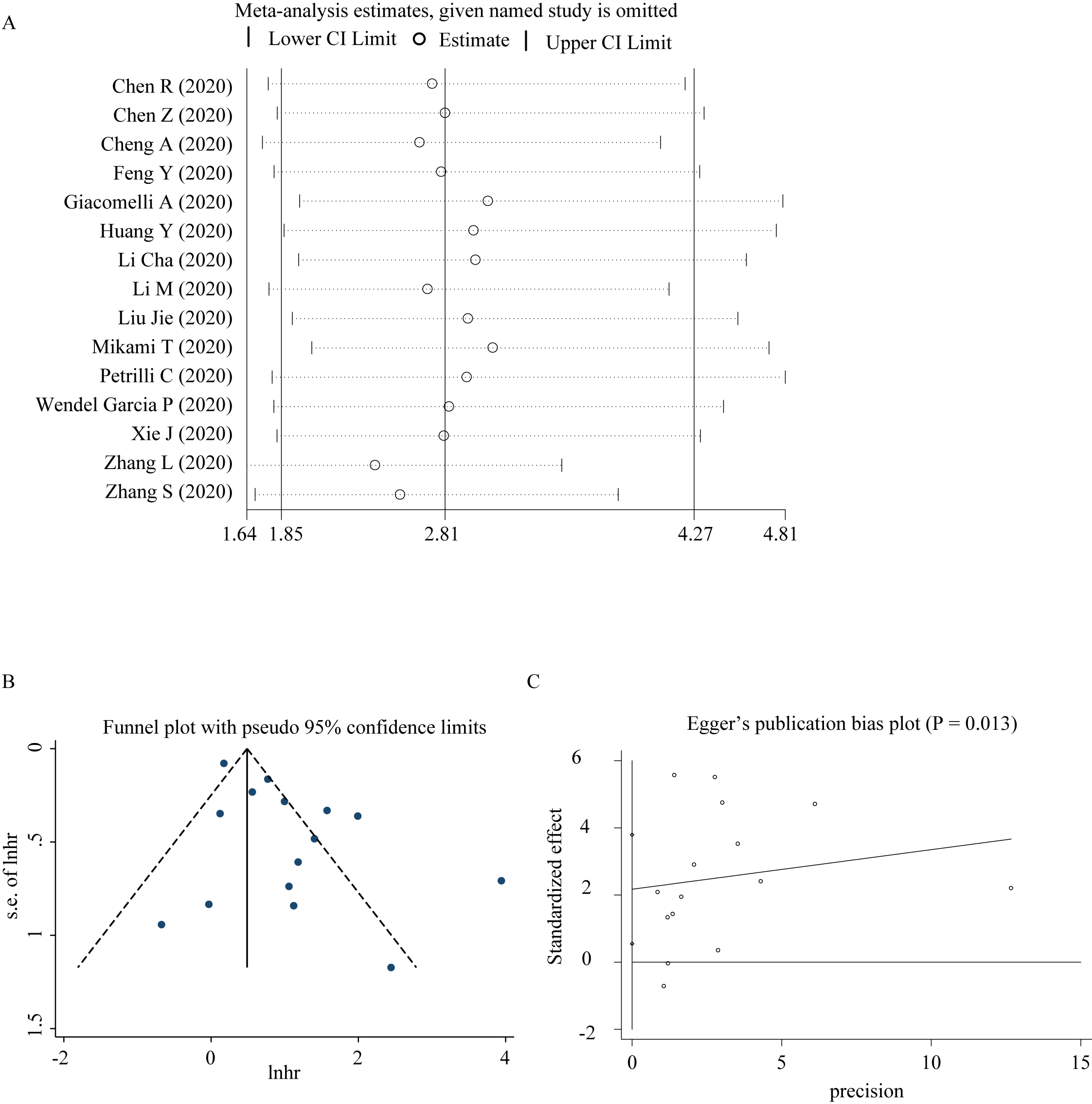

Supplement: Supplementary Figure 3 — Sensitivity analyses (A), funnel plot (B) and Egger test (C) of HR of COVID-19 mortality for D-dimer. [file Image_3.TIF]
