# Supplementary material for: Clinical Significance of Plasma D-Dimer in COVID-19 Mortality
Source: Front Med (Lausanne). 2021 May 25;8:638097. doi: 10.3389/fmed.2021.638097 (PMC8185282; doi:10.3389/fmed.2021.638097)
Supplement: Supplementary Table 1 — Methodological quality of studies included in the meta-analysis. [file Table_1.docx]

**Table S1. Methodological Quality of Studies Included in the Meta-analysis.**

| First author | Year | Selection | Comparison | Exposure/Outcome | Total score |
| --- | --- | --- | --- | --- | --- |
| Aloisio E | 2020 | ☆☆☆☆ | - | ☆☆ | 7 |
| Ayanian S | 2020 | ☆☆☆☆ | - | ☆☆ | 7 |
| Bahl A | 2020 | ☆☆☆☆ | - | ☆☆ | 7 |
| Barman H | 2020 | ☆☆☆☆ | ☆ | ☆☆ | 8 |
| Bazzan M | 2020 | ☆☆☆ | - | ☆☆ | 6 |
| Berenguer J | 2020 | ☆☆☆☆ |  | ☆☆ | 7 |
| Berger J | 2020 | ☆☆☆☆ | ☆☆ | ☆☆ | 9 |
| Bhargava A | 2020 | ☆☆☆☆ | - | ☆☆ | 7 |
| Cao J | 2020 | ☆☆☆☆ | - | ☆☆ | 7 |
| Chen R | 2020 | ☆☆☆☆ | - | ☆☆ | 7 |
| Chen Ti | 2020 | ☆☆☆☆ | - | ☆☆ | 7 |
| Chen Ta | 2020 | ☆☆☆☆ | - | ☆☆ | 7 |
| Chen X | 2020 | ☆☆☆☆ | - | ☆☆ | 7 |
| Chen Z | 2020 | ☆☆☆☆ | - | ☆☆ | 7 |
| Cheng A | 2020 | ☆☆☆☆ | - | ☆☆ | 7 |
| Chilimuri S | 2020 | ☆☆☆☆ | ☆☆ | ☆☆ | 9 |
| Cortés-Tellés A | 2020 | ☆☆☆☆ | - | ☆☆ | 7 |
| Du R | 2020 | ☆☆☆☆ | - | ☆☆ | 7 |
| Feng Y | 2020 | ☆☆☆☆ | ☆ | ☆☆ | 8 |
| Giacomelli A | 2020 | ☆☆☆☆ | ☆ | ☆☆ | 8 |
| Guisado-Vasco P | 2020 | ☆☆☆☆ | ☆☆ | ☆☆ | 9 |
| Huang Y | 2020 | ☆☆☆☆ | ☆ | ☆☆ | 8 |
| Laguna-Goya R | 2020 | ☆☆☆☆ | - | ☆☆ | 7 |
| Li Cha | 2020 | ☆☆☆☆ | ☆ | ☆☆ | 8 |
| Li Che | 2020 | ☆☆☆☆ | - | ☆☆ | 7 |
| Li K | 2020 | ☆☆☆☆ | - | ☆☆ | 7 |
| Li L | 2020 | ☆☆☆☆ | - | ☆☆ | 7 |
| Li M | 2020 | ☆☆☆☆ | ☆ | ☆☆ | 8 |
| Li Y | 2020 | ☆☆☆☆ | ☆ | ☆☆ | 8 |
| Liao D | 2020 | ☆☆☆☆ | ☆☆ | ☆☆ | 9 |
| Liu Jie | 2020 | ☆☆☆☆ | ☆ | ☆☆ | 8 |
| Liu Jia | 2020 | ☆☆☆☆ | - | ☆☆ | 7 |
| Lu J | 2020 | ☆☆☆☆ | - | ☆☆ | 7 |
| Luo X | 2020 | ☆☆☆☆ | - | ☆☆ | 7 |
| Ma X | 2020 | ☆☆☆☆ | - | ☆☆ | 7 |
| Manocha K | 2020 | ☆☆☆☆ | - | ☆☆ | 7 |
| Mikami T | 2020 | ☆☆☆☆ | ☆ | ☆☆ | 8 |
| Musoke N | 2020 | ☆☆☆☆ | ☆☆ | ☆☆ | 9 |
| Pan F | 2020 | ☆☆☆☆ | - | ☆☆ | 7 |
| Paranjpe I | 2020 | ☆☆☆☆ | - | ☆☆ | 7 |
| Peng X | 2020 | ☆☆☆☆ | - | ☆☆ | 7 |
| Petrilli C | 2020 | ☆☆☆☆ | - | ☆☆ | 7 |
| Piñana J | 2020 | ☆☆☆☆ | - | ☆☆ | 7 |
| Qin Z | 2020 | ☆☆☆☆ | - | ☆☆ | 7 |
| Quintana-Díaz M | 2020 | ☆☆☆☆ | ☆ | ☆☆ | 8 |
| Singh N | 2020 | ☆☆☆☆ | - | ☆☆ | 7 |
| Song K | 2020 | ☆☆☆☆ | - | ☆☆ | 7 |
| Tu W | 2020 | ☆☆☆☆ | - | ☆☆ | 7 |
| Volo T | 2020 | ☆☆☆☆ | - | ☆☆ | 7 |
| Wang K | 2020 | ☆☆☆☆ | - | ☆☆ | 7 |
| Wang L | 2020 | ☆☆☆☆ | - | ☆☆ | 7 |
| Wendel Garcia P | 2020 | ☆☆☆☆ | ☆ | ☆☆ | 8 |
| Xia P | 2020 | ☆☆☆☆ | - | ☆☆ | 7 |
| Xie J | 2020 | ☆☆☆☆ | ☆ | ☆☆ | 8 |
| Xu P | 2020 | ☆☆☆☆ | - | ☆☆ | 7 |
| Yang C | 2020 | ☆☆☆☆ | ☆ | ☆☆ | 8 |
| Yang K | 2020 | ☆☆☆☆ | - | ☆☆ | 7 |
| Yao Q | 2020 | ☆☆☆☆ | - | ☆☆ | 7 |
| Yao Y | 2020 | ☆☆☆☆ | ☆ | ☆☆ | 8 |
| Yu C | 2020 | ☆☆☆☆ | ☆☆ | ☆☆ | 9 |
| Zhang J | 2020 | ☆☆☆☆ | - | ☆☆ | 7 |
| Zhang L | 2020 | ☆☆☆☆ | - | ☆☆ | 7 |
| Zhang S | 2020 | ☆☆☆☆ | - | ☆☆ | 7 |
| Zhou F | 2020 | ☆☆☆☆ | ☆ | ☆☆ | 8 |
| Zhou J | 2020 | ☆☆☆☆ | ☆☆ | ☆☆ | 9 |
| Zhou S | 2020 | ☆☆☆☆ | - | ☆☆ | 7 |
